# Altered Functional Connectivity within and between Brain Modules in Absence Epilepsy: A Resting-State Functional Magnetic Resonance Imaging Study

**DOI:** 10.1155/2013/734893

**Published:** 2013-09-26

**Authors:** Cui-Ping Xu, Shou-Wen Zhang, Tie Fang, Ma Manxiu, Qian Chencan, Chen Huafu, Hong-Wei Zhu, Yong-Jie Li, Liu Zuxiang

**Affiliations:** ^1^Department of Functional Neurosurgery, Xuanwu Hospital, Capital Medical University, Beijing 100053, China; ^2^State Key Laboratory of Brain and Cognitive Science, Institute of Biophysics, The Chinese Academy of Sciences, 15 Datun Road, Chaoyang District, Beijing 100101, China; ^3^University of Chinese Academy of Sciences, Beijing 100039, China; ^4^Key Laboratory for NeuroInformation of Ministry of Education, School of Life Science and Technology, University of Electronic Science and Technology of China, Chengdu 610054, China

## Abstract

Functional connectivity has been correlated with a patient's level of consciousness and has been found to be altered in several neuropsychiatric disorders. Absence epilepsy patients, who experience a loss of consciousness, are assumed to suffer from alterations in thalamocortical networks; however, previous studies have not explored the changes at a functional module level. We used resting-state functional magnetic resonance imaging to examine the alteration in functional connectivity that occurs in absence epilepsy patients. By parcellating the brain into 90 brain regions/nodes, we uncovered an altered functional connectivity within and between functional modules. Some brain regions had a greater number of altered connections and therefore behaved as key nodes in the changed network pattern; these regions included the superior frontal gyrus, the amygdala, and the putamen. In particular, the superior frontal gyrus demonstrated both an increased value of connections with other nodes of the frontal default mode network and a decreased value of connections with the limbic system. This divergence is positively correlated with epilepsy duration. These findings provide a new perspective and shed light on how functional connectivity and the balance of within/between module connections may contribute to both the state of consciousness and the development of absence epilepsy.

## 1. Introduction

Absence epilepsy is characterized by brief episodes of impaired consciousness that last 2–10 s and that are associated with bursting bilateral and symmetrical electroencephalography (EEG) discharges consisting of 2.5–5 Hz spike-and-wave complexes on a normal background [1]. These spike-and-wave discharges (SWDs) begin and end abruptly [2], and the accompanying behavioral changes have been thoroughly investigated [3]. A model suggests that these discharges may be generated by oscillations between the thalamus and a slightly hyperexcitable cortex and depend on long-range corticothalamic and corticocortical network interactions [4]. This model has been supported by previous studies which combined functional magnetic resonance imaging (fMRI) with simultaneous EEG recordings [5] and found an increased bilateral fMRI BOLD response in the thalamus and decreases in cortical areas (including the frontal and parietal cortices).

In addition to the altered activations related to SWDs, previous studies have demonstrated that absence epilepsy patients also exhibit anatomical changes such as an increased volume in the anterior thalamus [6], an increase in gray matter concentration in the superior mesiofrontal region [7], and smaller gray matter volumes of the left orbital frontal gyrus and both the left and right temporal lobes [8]. Diffusion tensor imaging studies have revealed that there are fiber changes associated with absence epilepsy in an animal model [9] that have been confirmed in human patients by the observation of increased mean diffusivity (MD) values bilaterally in thalamus, putamen, and left caudate nucleus and increased fractional anisotropy (FA) values in bilateral caudate nuclei [10]. These results suggest an important role for functional connections between cortical areas and subcortical regions in absence epilepsy. Additional indirect evidence to support this assumption comes from a case report of a 4.5-year-old girl with periventricular nodular heterotopia that induced typical absence seizures, probably because the heterotopia influenced the formation and excitability of the striatothalamocortical network [11].

Resting-state fMRI can be used to measure the temporal correlations of blood oxygen level-dependent (BOLD) signals in distant brain regions and is presumed to reveal spontaneous, intrinsic, and functional connectivity. This technique has been demonstrated to be useful in exploring multiple brain functional networks, such as vision, language, attention, and the default mode network (DMN) [12] and has also been used to compare normal subjects with patients with neuropsychiatric disorders [13]. The use of resting-state fMRI provides a good opportunity to directly explore the corticothalamic network in absence epilepsy by examining its functional connectivity. A closely related study that utilized this approach investigated the relationship between absence epilepsy (AE) and the DMN by exploring the changes of functional connectivity in AE patients [14]. These authors defined DMN nodes by using the posterior cingulated cortex (PCC) as a seed to determine the resting-state functional connectivity and found that several links of the DMN have a decreased functional connectivity in AE patients when compared with control subjects. However, a limitation of this study was that only nodes in the DMN were evaluated; therefore, any possible alterations of connections within and between different brain modules, such as attention networks or the somatosensory system, would be excluded.

Instead of defining relevant functional nodes by a seeded region of interest (ROI), an approach commonly used in network analysis studies is to parcellate the brain into multiple regions/nodes by a previously defined automatic template [15]. These nodes form distinct functional modules that can be revealed by resting-state functional connectivity and fit well with other task-involved fMRI results [16]. It has been demonstrated that the characteristics of the functional modules have been altered in mental disorders, such as schizophrenia [17].

In this study, we use the predefined brain mask to study functional connectivity in AE patients instead of defining the ROI as the seed. We compared the differences of functional connectivity pattern between AE patients and normal control groups. We also explore the relations of different brain modules in AE patients in the context of rest-state conditions.

## 2. Methods

### 2.1. Subjects

We recruited a total of 12 patients (20.0 ± 10.5 years old, ranging from 8 to 40 years; 5 males and 7 females) with AE from XuanWu Hospital in Beijing. All patients underwent a clinical MRI to measure their brain structure and long-term video EEG monitoring. No patient exhibited any radiological abnormalities. A diagnosis of AE was established according to the diagnostic scheme published by the International League Against Epilepsy in 2001 [18], and all patients had typical electroclinical features of AE at the time of the diagnosis. These patients experienced absence seizures anywhere from 1-2 times a year to 5–10 times a day (see Table 1, reported by relatives). For the 5 patients with only absence seizures without any other syndromes, no medications were taken before the diagnosis and the fMRI scans. No patient reported absence seizure during the scans. After the experiment, the MRI operator talked with each patient and confirmed it. All the patients then had been prescribed valproate sodium, lamotrigine, or levetiracetam after the fMRI scans. A separate group of 14 normal subjects (22–28 years, 24.6 ± 1.5 years old, 8 males and 6 females) were selected as controls. All control subjects were healthy and free from any history of epilepsy, neurological disorders, or other major medical illness. All patients and healthy subjects gave informed consent before the experiment. The consent and experimental procedures were proved by the XuanWu Hospital Institutional Review Board.

### 2.2. Data Acquisition

The functional MRI scan was conducted with a Siemens Tim 3T system (Siemens Medical Solutions, Erlangen, Germany). BOLD responses were acquired using single-shot, gradient-echo, echo-planar imaging (EPI) with the following parameters: FOV = 192 mm, TR = 2000 ms, TE = 30 ms, flip angle = 90 degrees, and voxel size = 3 × 3 × 3.5 mm^3^. The functional scans consisted of 33 axial slices that covered the whole brain, and each scan had 240 functional volumes. Two runs were acquired for each patient or control subject. A rapid anatomical scan that had the same center and slice orientation as the functional scans was obtained using the magnetization-prepared rapid acquisition gradient-echo (MP-RAGE) sequence with the following parameters: TR = 810 ms, TE = 2.39 ms, flip angle = 15 degrees, voxel size = 1.1 × 0.8 × 2 mm^3^, and 144 axial slices. An anatomical scan with an isotropic resolution was obtained using the same MP-RAGE sequences with different parameters: TR = 2600 ms, TE = 3.02 ms, flip angle = 8 degrees, voxel size = 1 × 1 × 1 mm^3^, and 176 sagittal slices. 

### 2.3. Functional Connectivity

Only the second functional scan was used for the data analysis because of the more stabilized resting state in the subjects. The preprocessing of the fMRI data involved a MATLAB (Mathworks, Natick, MA, USA) toolbox called Data Processing Assistant for Resting-State fMRI (DPARSF) [19]. All 240 time points in the functional scan were included in the analysis. The data were first corrected for slice timing and head motion before being normalized into Montreal Neurological Institute (MNI) space using the default EPI templates in the toolbox. The data were then smoothed with the default full width at half-maximum (FWHM) parameter set as 4 mm.

After detrending and filtering with a default value of 0.01–0.08 Hz, the data were parcellated into 90 cortical and subcortical regions using an already established anatomical automatic labeling template [15] provided with the toolbox. Nuisance covariates including 6 head motion parameters, whole brain, white matter, and cerebrospinal fluid signals were regressed out before a calculation was made to determine the functional connectivity matrix of the 90 regions.

A 90 × 90 functional connectivity matrix was generated for each subject (control or patient). An independent *t*-test was performed for each entry of the functional connectivity matrix to determine the difference between the AE and control groups. A threshold of *P* < 0.01 (FDR corrected) was established to generate the difference matrix, in which an entry is set to 1 if the AE group has a significantly larger functional connectivity than the control group or −1 if functional connectivity of the AE group is significantly smaller than the control group.

### 2.4. Module Definition and Visualization

**Figure 1:**
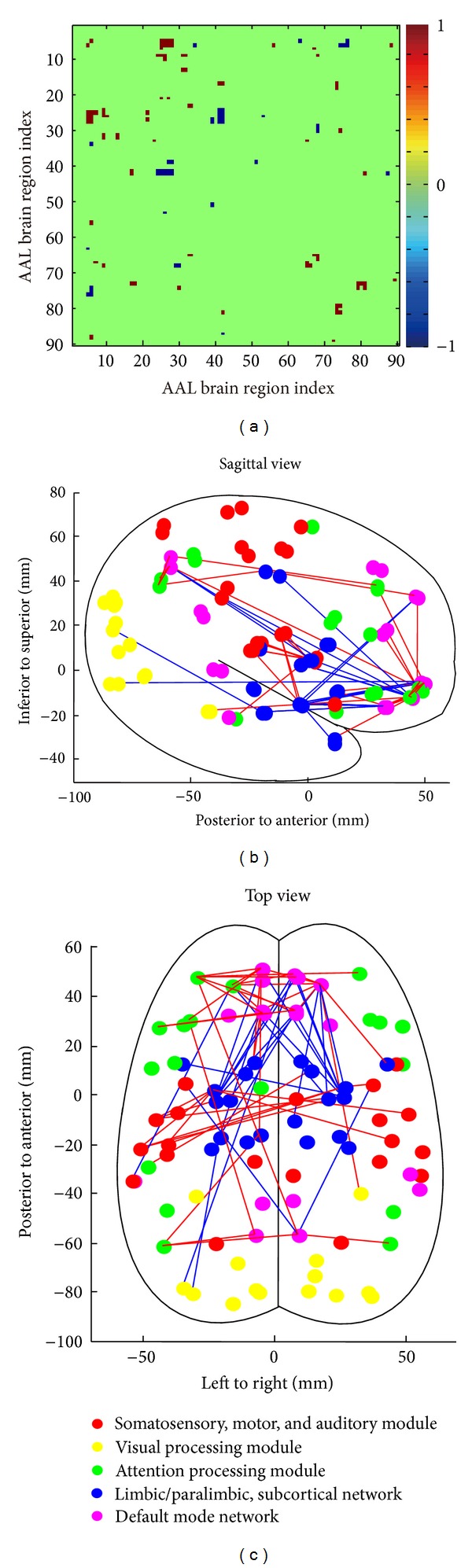
Altered functional network between AE and control. (a) Significant differences between the AE and control groups were found (*P* < 0.01). For links that showed larger correlation values in the AE group, the corresponding cells of the matrix were set to 1, while smaller correlation values in the AE group resulted in a −1 value for the corresponding cells of the matrix; those cells without a significant difference had a value of 0. (b) A lateral view and (c) a top view of this network are shown. All of the 90 brain regions are marked, and the different colored spheres represent distinct network modules. Notably, the regions are localized according to their centroid stereotaxic coordinates. The edges between the nodes were constructed based on the binarized difference matrix; the red edges indicate that the AE group has a larger functional connectivity than normal, and the blue edges indicate a decreased functional connectivity in the AE group.

The 90 brain regions were separated into 5 modules: I, somatosensory, motor, and auditory; II, visual processing; III, attention processing; IV, default mode network; and V, limbic/subcortical system [20]. Different node colors were used for each module (Figure 1). The coordinates of the center of mass for each brain region were used to lay out the 90 nodes in 3D space in MATLAB using a previously described method [20]. The edges between the nodes were constructed on the basis of the differences in the correlation matrix between AE and control subjects; red edges indicate that the AE group has a larger functional connectivity than normal and blue edges represent a decreased functional connectivity in the AE group.

### 2.5. Identification of the Important Nodes

To examine the importance of the nodes, the number of altered connections of each region/node was examined as a node-connection distribution. The resulting histogram showed that most nodes have only 1 or 2 altered connections and that only a few nodes have multiple altered connections. The mean and standard deviation were calculated for this node-connection distribution, and a threshold of mean + 2.326 standard deviation was used to select the nodes with the greatest number of altered connections. To correct for the multicomparison problem for this test, a 1000-round permutation procedure was applied. The distribution and histogram of the node-connections were generated, and the value of mean + 2.326 standard deviation for each permutation was determined (see Figure S1A in Supplementary Material available online at http://dx.doi.org/10.1155/2013/734893). This method allowed for a multicomparison correction at the threshold of *P* < 0.001 to identify the most important nodes (Figure S1B).

For each selected node (with the greatest number of altered connections), an average value of the altered functional connections within the module and between modules was calculated; this average was calculated from the original correlation matrix (*r* value, instead of the binarized difference matrix) for each patient and each control subject. A mixed, repeated-measures general linear model (GLM) was used for further statistical analysis in which relation to modules (within versus between modules) was defined as a within-subject factor, and subject group (AE versus control) was defined as a between-subject factor.

### 2.6. Differential Connectivity

As one of the frontal nodes in the DMN module, the superior frontal gyrus (specifically the orbital region) showed more positive connections with other frontal nodes of the DMN (within module) in patients than control subjects; this same area also demonstrated more negative connections to the limbic system (between modules). A differential connectivity was defined to index this divergence within and between modules as follows:

(1) Differential  connectivity =|connections  to  other  frontal  DMN  nodes|  +|connections  to  limbic  system|.

The relationship between the differential connectivity and the clinical variables (i.e., age, initial age of seizure onset, epilepsy duration, and seizure frequency) was tested using Pearson's correlation at a threshold of *P* < 0.05.

For other two nodes found to be important and had similar divergence between modules, similar differential connectivity was calculated, and relationship to clinical variables was also evaluated.

## 3. Results

### 3.1. Altered Functional Connectivity between the AE Group and the Control Group

A significantly altered functional connectivity matrix was constructed by comparing the difference between the AE group and the control group for each entry in the functional connectivity matrix with an independent *t*-test at a threshold of *P* < 0.01 (FDR corrected, Figure 1(a)). The entries in red indicate that the AE group showed a significantly larger functional connectivity than the control group in those links, while the entries in blue indicate that the AE group showed a significantly smaller functional connectivity than the control group.

To achieve a more comprehensive understanding of the results, the matrix was visualized in a 3D space from both sides and top views (Figures 1(b) and 1(c)), and 90 brain region nodes were divided into 5 differently colored modules. An examination of the results (Figures 1(b) and 1(c)) clearly reveals that the AE group has a decreased functional connectivity between nodes in the default mode network (DMN; pink nodes), specifically in nodes in frontal areas and the nodes in the limbic system (blue nodes). It is also significant that some nodes in the limbic system (the putamen and the amygdala) showed increased functional connections to sensorimotor systems (red nodes) in the AE group. If the value of the differences in each link with a threshold *P* < 0.0005 (uncorrected) is used to emphasize the severely altered connections to the nodes, a clearer pattern revealed that the frontal DMN nodes have increased connections to frontal nodes and decreased connections to limbic nodes (Figure S2).

### 3.2. Important Brain Regions with the Highest Altered Functional Connectivity

**Figure 2:**
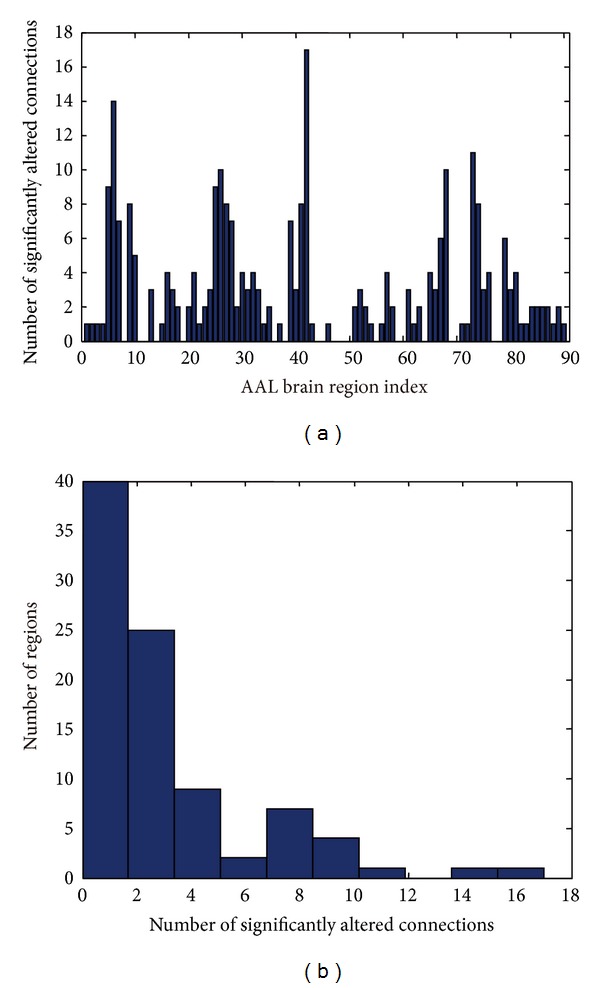
Statistical characteristics of significantly altered connections for each node/region. (a) The number of significantly altered connections was determined. It is clear that among the 90 nodes, most had fewer than 4 significantly altered connections, while some nodes have more than 10 significantly altered connections. (b) A histogram shows the distribution of the number of significantly altered connections among the 90 nodes. Importantly, only 3 nodes have more than 11 significantly altered connections, and there is a bump in the distribution above 6 connections.

**Figure 3:**
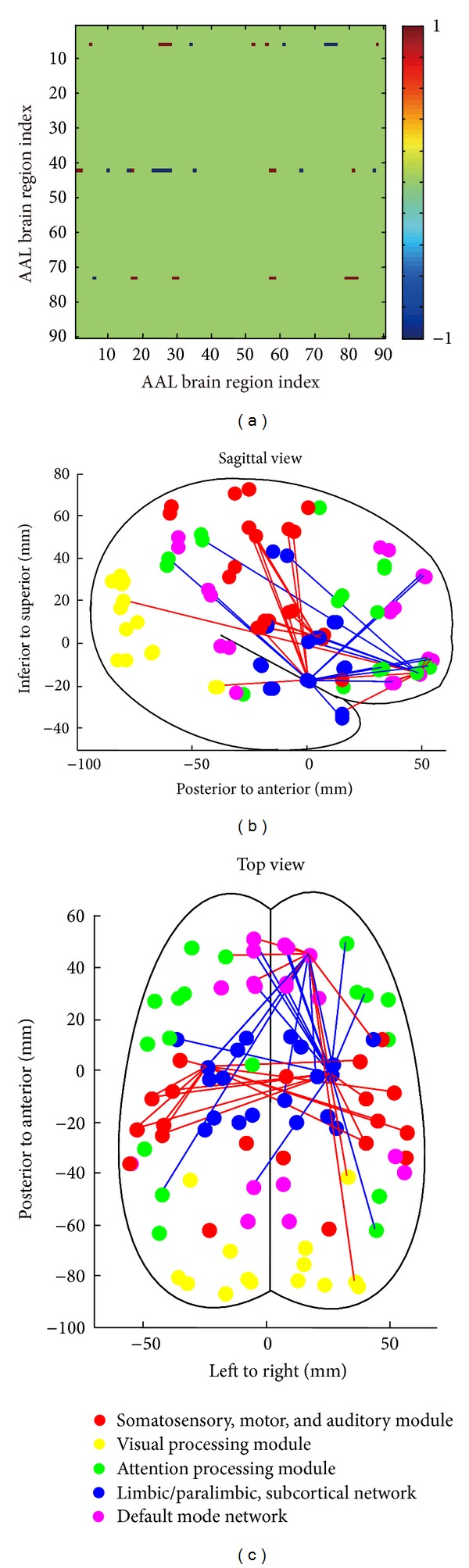
Network of 3 nodes with more than 11 significantly altered connections. (a) The 3 nodes with more than 11 significantly altered connections were identified. The binarized interregional correlation matrix was filtered to include only these 3 nodes and their altered connections. In (b) and (c), an anatomical network representation was constructed based on the filtered matrix from (a). Most of the structure and pattern of the matrix were retained after filtering.

The number of significantly altered connections among each of the 90 brain regions is illustrated in Figure 2(a). While the number of significantly altered connections for each node ranges from 0 to 17, only a small subset of regions had more than 4 altered connections. A histogram of the distribution (Figure 2(b)) confirmed that the number of nodes with each number of altered connections decreased until a value of 6 altered connections was reached. This distribution has a mean of 2.911 and a standard deviation of 3.364; therefore, a threshold of 11 altered connections was chosen to find the most important brain regions (at *P* < 0.001 level, see Figure S1). After applying this threshold, the three most important brain regions with at least 11 altered functional connections were identified as the superior frontal gyrus (orbital part), the right amygdala, and the left putamen (Figure 3(a)). It is interesting to note that the 3D graph (Figures 3(b) and 3(c)) constructed from these three nodes and their altered connections has almost the same key features as the original alerted functional connectivity matrix that is shown in Figure 1.

**Figure 4:**
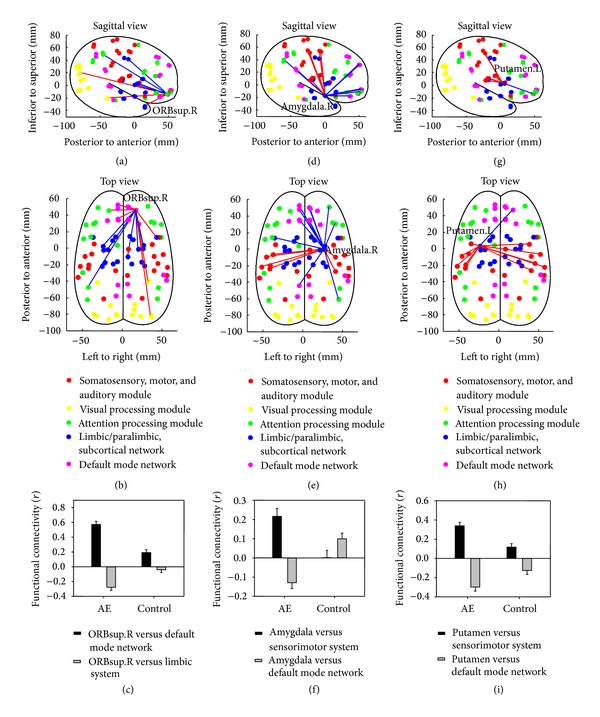
The pattern of within/between module divergence for individual nodes. (a) and (b) The orbital part of the superior frontal gyrus showed an increased within-module connection that is represented as an increase in the functional connections to other DMN nodes and a decreased between-module connection that is represented as a decrease in the functional connections to the limbic system in AE patients. (c) The pattern of divergence for the orbital part of the superior frontal gyrus indicates that the value of connections to other DMN nodes is already positive in controls but becomes even more positive in AE patients, while the value of connections to the limbic system is negative in controls and becomes even more negative in AE patients. This divergence showed a significant interaction of within/between module connections and subject groups (*P* < 0.0001). (d) and (e) The right amygdala showed an increased value of connections to the somatosensory system and a decreased value of connections to the DMN in AE patients. (f) The pattern of divergence revealed that the value of connections to the somatosensory system is almost zero in controls and increases in AE patients, while the value of connections to the DMN is positive in controls but decreased to a negative value in AE patients (*P* < 0.0001). (g) and (h) The left putamen showed an increased value of connections to the somatosensory system and a decreased value of connections to the DMN in AE patients. (i) The pattern of divergence showed that the number of connections to the somatosensory system is positive in controls but became even more positive in AE patients, while the value of connections to the DMN is negative in controls and became even more negative in AE patients (*P* < 0.0001); this pattern is identical to the previously observed pattern in the superior frontal node. ORBsup.R, superior frontal gyrus (right orbital); Amygdala.R, amygdala (right); Putamen.L, putamen (left).

As one of the frontal DMN nodes, the orbital part of the superior frontal gyrus showed a clear pattern of altered connections. The links to other default network nodes are stronger, while links to the limbic system are weaker in the AE group when compared with the control group (Figures 4(a) and 4(b)). The average value of these connections showed that while there are positive functional connections between this node and other frontal DMN nodes in the control group, AE patients have even more positive connections than normal; in contrast, while functional connections to the limbic system are negative or near zero in controls, AE patients have more negative connections (Figure 4(c)). A significant interaction (*F*(1,24) = 105.44, *P* < 0.0001) confirmed that the changes in the connections to the DMN nodes and the limbic system occur in the opposite direction.

The two limbic system nodes (the right amygdala and the left putamen) shared the same increase in links to the sensorimotor system and the same decrease in links to DMN nodes in AE patients (Figures 4(d), 4(e), 4(g), and 4(h)). An average value of these connections showed that connections to sensorimotor nodes become more positive and connections to DMN nodes become more negative in the AE group when compared with the control group (Figures 4(f), 4(i)). Significant interactions (*F*(1,24) = 35.45, *P* < 0.0001 and *F*(1,24) = 22.95, *P* < 0.0001, resp.) confirmed these observed differences.

### 3.3. The Relationship between Differential Connectivity and Clinical Features

**Figure 5:**
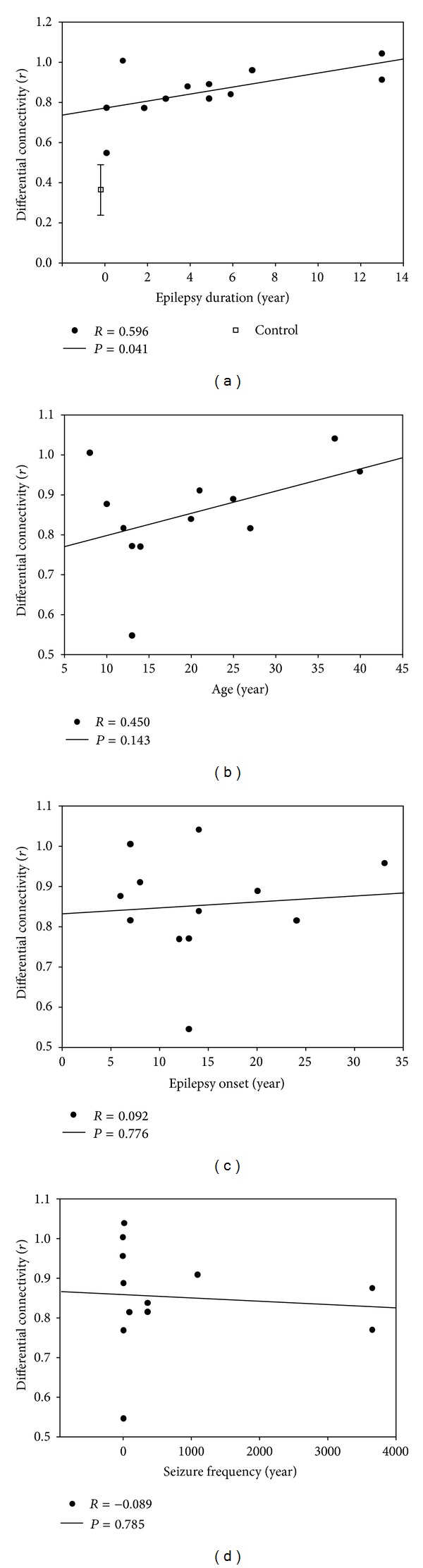
The relationship between differential connectivity and epilepsy. (a) The divergence of the superior frontal gyrus (indexed as differential connectivity) showed a significant correlation with the duration of epilepsy; the longer the patient has suffered from epilepsy, the higher the differential connectivity becomes. (b) Age showed a positive trend with the divergence, but the relationship is not significant. (c) Epilepsy onset and (d) seizure frequency showed no significant correlation with the divergence.

Differential connectivity was computed for the three previously mentioned brain regions to describe the within/between module changes. A significant correlation between the differential functional connectivity of the orbital part of the superior frontal gyrus and the duration of epilepsy was found (*R* = 0.596, *P* = 0.041; Figure 5(a)). It is interesting to note that this differential connectivity increased with epilepsy duration, while control subjects that lack epileptic seizures showed a smaller differential connectivity which was well below that of the AE patients (*t*(24) = 3.06,  *P* < 0.01). Age was found to have a positive trend similar as epilepsy duration (Figure 5(b)). Anyhow, the relationship is not significant (*R* = 0.450, *P* = 0.143). No significant correlation was observed between the differential functional connectivity and the initial age of seizure onset (*R* = 0.094, *P* = 0.776) or seizure frequency (*R* = −0.089, *P* = 0.785). The other two limbic nodes did not show any significant correlation with these variables.

To address the possibility that the previously mentioned frontal-limbic correlation is only valid for the orbital part of the superior frontal gyrus due to the specific function of this brain region and therefore cannot be generalized to other frontal DMN nodes, we lowered the threshold of importance to nodes with at least 6 altered connections. As shown in Figure S3, the constructed matrix has more nodes remaining, and the graph has the same pattern as Figure 3. For all three frontal DMN nodes that meet this threshold, the within-DMN connections are still larger, and links to the limbic system are again smaller in the AE group compared with the control group (Figure S4); this pattern is identical to the one shown in Figures 4(a) and 4(b). The average value of these connections reinforced the results from Figure 4(c) where within-DMN links were found to be more positive than normal, and links to the limbic system were found to shift in the opposite direction, that is, more negative than normal (Figure S5, *F*(1, 24) = 72.119, *P* < 0.0001). The correlation between the differential functional connectivity of these frontal nodes and the duration of epilepsy is also significant (Figure S6, *R* = 0.872, *P* = 0.0002), and the differential connectivity of the control group is significantly smaller than that of the AE group (*t*(24) = 6.057, *P* < 0.001).

## 4. Discussion

In this study, we found that AE patients demonstrated an alteration in several functional links and that there was a significant divergence of functional connectivity within and between brain modules. In AE patients, nodes in the limbic system, including the amygdala and the putamen, showed decreased functional connections to the DMN; in contrast, the connections to somatosensory system were increased. We also found that frontal nodes of the DMN showed increased connections within the DMN module and decreased connections to limbic system of AE patients. This within/between module divergence of frontal nodes was positively correlated with the duration of epilepsy.

### 4.1. Altered Interregional Functional Connectivity

Using a predefined anatomical-based template, the resting-state data collected by fMRI were parcellated into 90 node/regions. Interregional correlation (i.e., functional connectivity) showed that, for the AE patients, there are stronger connections between frontal DMN nodes and other frontal nodes as well as between posterior DMN nodes and frontal/parietal nodes. These data conflict with a previous study that found a decreased functional connectivity in the DMN for AE patients [14]. However, the current results are facilitated by a different approach compared to the previous study. While the DMN in the previous study was defined by the functional connectivity that was seeded at the posterior cingulated cortex (PCC), the brain regions/nodes were automatically defined by an anatomical template in the current study. The modules of these nodes were defined by a network study [20]. We believe that this approach could provide more sensitive and detailed information about the alteration in functional networks. In addition, if we only considered the links from both sides of the PCC, we found a similar decrease in connections to the right temporal cortex as was observed by Luo et al. [14], although the links to left frontal cortex are increased in AE patients. The inconsistency between the current results and the previous study may indicate that different dynamics exist within frontal and posterior DMN regions if they are parcellated into subregions. However, the brain regions/nodes revealed by the altered connections, including thalamic regions and the frontal and parietal cortices, are consistent with previous EEG/fMRI results obtained from AE patients.

We also found an increased functional connectivity to the somatosensory system that primarily originated from the limbic system. Because somatosensory regions were not obvious regions of interest when the DMN was defined by seeding at the PCC, the current results showed for the first time how the interaction between the DMN and other functional modules is altered in AE patients. The increased connection to the somatosensory system fit well with the observation that after, stimulation, the response of somatosensory cortex is heightened and followed by SWD-like afterdischarges (ADs) in animals with AE [21]. The SWD-like ADs are assumed to indicate changes in corticothalamocortical network, which is directly demonstrated in the current study. If we consider the increased within-frontal DMN connections together with this increased connections from subcortical structures to the somatosensory system, these results may provide support for the idea that a slightly hyperexcitable cortex is one of the main factors of AE [22].

Another significant feature of the altered network is the decreased connections from the limbic system. This study is the first to observe the functional relationship between subcortical structures and cortical areas in absence epilepsy. The results are somewhat unexpected because in most EEG/fMRI studies, the SWD-triggered activation of thalamic areas is increased, while the activation in cortical areas is decreased [23]. Anyhow, the changes in SWDs that were revealed by the EEG/fMRI study reflect the brain regions that are directly involved in seizure discharges, while the current result is based on resting-state fMRI and therefore focuses on alterations that occur in the absence of seizures. These functional or anatomical changes that occur during periods where the patients do not exhibit seizures may contribute to our understanding of the underlying mechanism of absence epilepsy and its dynamics during seizure development. This difference suggests that subcortical and cortical areas each play a different role in the SWD dynamics of absence seizures. The current results showed that the thalamic and cortical areas also have different functional dynamics even under resting-state conditions. This finding adds some novel information to the long-debated field of SWD generation in thalamocortical networks [24]. It suggests that the decreased functional connections between subcortical and cortical areas under resting-state conditions should be incorporated into the debate. 

### 4.2. Divergence of within/between Module Connections in AE Patients

We identified 3 brain nodes/regions that showed a significantly higher number of altered connections after a multicomparison correction was applied. The connections from these nodes showed almost the same patterns as the original network, suggesting that these three nodes may behave as hubs in the network analysis [25]. More important, these nodes showed an increased divergence of within/between-module connections, which were defined as a differential functional connectivity. This divergence has clinical implications in that for the superior frontal gyrus, it is positively correlated with epilepsy duration. This result is the first in human patients to demonstrate that the balance between functional connectivity and within/between brain modules plays a role in the development of absence epilepsy. Anatomical changes that are related to absence epilepsy may contribute to this divergence. This idea is supported by previous findings. Some studies have shown that the volume and concentration of gray matter increase in some cortical regions and thalamus but decrease in others [8], while other studies have revealed changes in the diffusion parameters of subcortical nuclei [10] and a lower thalamic NAA/Cr ratio in absence epilepsy patients [26]. Several animal model studies may help to reconcile these results. Using WAG/Rij and GAERS rats, a diffusion tensor image (DTI) scan revealed a decreased FA value in the anterior corpus callosum. This change was related to SWD onset, and the decrease in FA was induced by an increased perpendicular diffusivity [9]. Although this study did not directly demonstrate any changed fiber connections between subcortical and cortical brain areas, it suggested that fiber changes related to absence seizures do occur and that these changes are positively correlated with epilepsy discharges. After cortical stimulation, the lowest threshold was observed for the transition to the limbic type of after discharges in WAG/Rij rats [27], which indicated that the connection between the cortex and the limbic system has been altered to be more sensitive in these animals. By recording local field potentials in this animal model, it has been suggested that the thalamus and cortical regions have different temporal dynamics [4] and increased coherence [28] during SWD onset. However, it would be particularly interesting to test whether the temporal dynamics of the thalamus and cortical regions are different during resting states. Another important factor that could be involved in this divergence is the distribution of neurotransmitters throughout different parts of the brain; the distribution of the D_2_-like dopamine receptor has been found to change in AE rats [29]. In addition to fiber changes that have been revealed by diffusion tensor imaging [10], changes in neurotransmitter distribution could be directly related to the breakdown of the balance of within/between-module functional connectivity.

However, we also noticed that there is a confounding factor in our AE group. The older the patient is, the longer epilepsy duration he/she has, in most cases. As shown in Figure 5(b), the divergence of functional connectivity also has a positive trend with age in patients, though the correlation between them is not significant. Due to the intrinsic connection in epilepsy duration and age, we could not rule out the influence of age in our AE group. So the relationship between this divergence and epilepsy duration should be regarded as very preliminary. Further study with large population of patients is required to address this possibility. Anyhow, with a more age-matched group (see Supplementary Methods), we confirmed that the differences and the within-between-module pattern between AE patients and control subjects could not be simply explained by the age of AE patients (Figure S7). More importantly, there is no significant correlation found between this within-between-module divergence and age, in the age-matched control subjects (Figure S8).

### 4.3. The Relationship of Functional Connectivity to Consciousness

The role of the DMN that has been revealed by functional connectivity studies is assumed to correlate with states of consciousness in epilepsy [14], although some evidence has suggested that slow, coherent, and spontaneous BOLD fluctuations cannot exclusively be a reflection of conscious mental activity [30]. In this study, we found that the superior frontal gyrus (a frontal DMN node) exhibited increased within-DMN connectivity and a decreased connectivity to the limbic systems. More important, this divergence is positively correlated with epilepsy duration. This finding could shed a light on the relationship between functional connectivity in epilepsy and consciousness and the possible role of the DMN.

Our current idea about the contribution of the DMN to consciousness is based on a popular theory about consciousness called the global workspace theory [31]. This theory proposes that widely distributed brain regions, especially frontoparietal associative cortices, should work coherently. However, the role of the limbic system or subcortical structures is seldom considered. By recording from a depth electrode that extends to thalamus, a previous study has revealed that impaired consciousness during temporal lobe seizures is related to increased long-distance cortical-subcortical synchronization [32]. It is possible that the sudden alterations in consciousness that are associated with epilepsy occur because of a nonlinear increase of neural synchrony within distant corticocortical and corticothalamic networks [33]. An explanation for this effect could be that the increased synchrony during epilepsy prevents the network from reaching the minimal level of differentiation and complexity that is necessary to code conscious representations. 

Initially, it seems that our observation of a decreased thalamic frontal connection in the AE group is inconsistent with the previous study. Fortunately, the method of calculation of synchrony allows that the increased synchrony could range either from 0 to 1 or 0 to −1; therefore, our finding that the decreased functional connectivity is more negative when compared with controls could still represent an increase in synchrony. Our data are also consistent with a study that found a propofol-induced decrease in consciousness [34]. That study also found that consciousness in control subjects linearly correlates with decreased corticocortical and thalamocortical connectivity in frontoparietal networks (i.e., default and executive-control networks). It is still unclear why the connection between the limbic system and the DMN needs to decrease while synchrony increases, because an increase in functional connectivity also leads to increased synchrony. One possible explanation is that because the connection between the limbic system and the DMN is already negative in control subjects, the epilepsy-induced breakdown of this balance, especially with regard to the increased sensitivity of the cortical-thalamic connection [27], is more fragile and more likely to move in the direction of a more negative connection. While this phenomenon may help us to understand the neural mechanisms of consciousness in general, further investigations will be required to identify the specific mechanisms in future studies.

There are several limitations in this study that we should be aware of. The sample size of patients is small. Though we found significant correlation between the change of functional connections and epilepsy duration, the results will be more general if we could obtain more patients in the further study. For patients with less seizure, the seizure frequency reported by relatives is very vague, without any solid evidence. Although we tested the relationship between seizure frequency and functional connectivity, we could not correct its influence on epilepsy duration in this paper. In further study, we will collect more long-term EEG with video recording, at least for patients with high seizure frequency, to make an accurate measure. Half of the patients in this study have absence epilepsy and generalized tonic-clonic seizure (GTCS). We monitored the status of the patients with a camera during the scan, and no GTCS was found. It is very unlikely that the current results are due to the GTCS, but the possibility remains that GTCS-induced long-term changes of functional connectivity were involved. It is also unclear if and how SWD will influence the within/between module network dynamics, a question should be addressed in the future with simultaneous EEG with fMRI and with new-onset epilepsy patients [35], from which the causality between the alteration of network connections and seizures could be resolved.

## Supplementary Material

A 1000-round permutation procedure was applied to correct for the multicomparison problem for the node-connection test. The distribution before and after sorting was presented in Figure S1. To confirm the pattern we found, we also present the differences between AE and normal with stricter threshold without multicomparison correction in Figure S2, or with smaller number of node-connections in Figure S3. Both of them showed similar patterns as the one described in the main text of the paper. To demonstrate that other frontal DMN nodes also showed the within-between-module diversity, the network generated by all frontal DMN nodes was presented in Figure S4 and the averaged value of functional connectivity was presented in Figure S5. The positive relations between epilepsy duration and within-between-module diversity is also true for all frontal DMN nodes, as shown in Figure S6. To exclude the possible confounds caused by the different age ranges of the AE and control group, data from another group of subjects age-matched to the AE patients were analyzed. We found a very similar pattern in the comparison of functional connection networks in Figure S7. In Figure S8, we also showed that the differential connectivity of orbital superior frontal gyrus is not significantly correlated with age in this control group.

## Figures and Tables

**Table 1:** Clinical information collected from the AE patients.

ID	Age (years)	Gender	Epilepsy duration (year)	Epilepsy subtype	Medication history	Seizure frequency	Medication after this test
1	12	F	5	AE	None	1-2/day	Lamotrigine
2	37	F	13	AE + GTCS	Carbamazepine	2-3/month	Levetiracetam
3	13	M	0^a^	AE	None	1-2/month	Valproate
4	10	M	4	AE + GTCS	Valproate	10/day	Valproate
5	8	F	0^b^	AE	None	4/year	Lamotrigine
6	25	F	5	AE + GTCS	Carbamazepine	1-2/month	Lamotrigine
7	14	M	2	AE + GTCS	Carbamazepine	1-2/month	Lamotrigine
8	40	F	7	AE + GTCS	None	2/year	Lamotrigine
9	20	M	6	AE + GTCS	Carbamazepine	1-2/day	Lamotrigine
10	21	F	13	AE	None	3–5/day	Lamotrigine
11	27	F	3	AE	None	2-3/week	Valproate
12	13	M	0^a^	AE + myoclonus	None	10/day	Levetiracetam

^a^3 months, ^b^7 months.
