# Supplementary figures and images for: Altered Functional Connectivity within and between Brain Modules in Absence Epilepsy: A Resting-State Functional Magnetic Resonance Imaging Study
Source: Biomed Res Int. 2013 Sep 26;2013:734893. doi: 10.1155/2013/734893 (PMC3804038; doi:10.1155/2013/734893)

**A**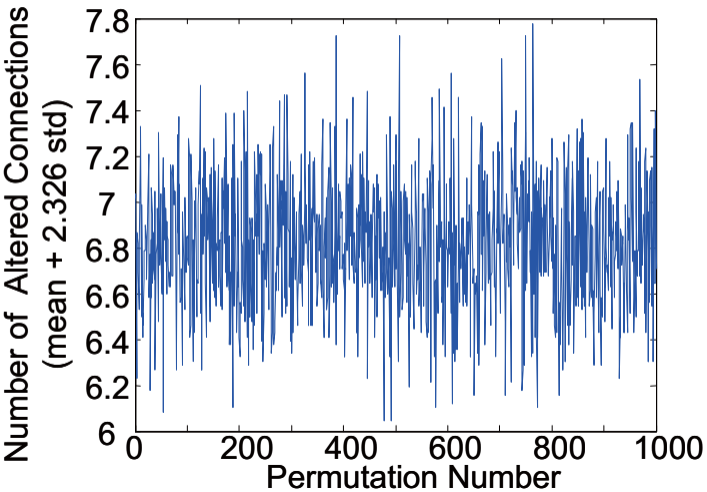**B**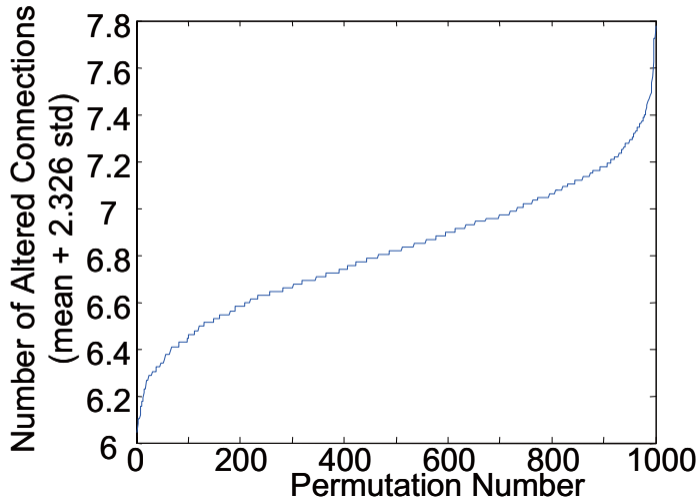

Supplement: Supplementary file 1 — A 1000-round permutation procedure was applied to correct for the multicomparison problem for the node-connection test. The distribution before and after sorting was presented in Figure S1. To confirm the pattern we found, we also present the differences between AE and normal with stricter threshold without multicomparison correction in Figure S2, or with smaller number of node-connections in Figure S3. Both of them showed similar patterns as the one described in the main text of the paper. To demonstrate that other frontal DMN nodes also showed the within-between-module diversity, the network generated by all frontal DMN nodes was presented in Figure S4 and the averaged value of functional connectivity was presented in Figure S5. The positive relations between epilepsy duration and within-between-module diversity is also true for all frontal DMN nodes, as shown in Figure S6. To exclude the possible confounds caused by the different age ranges of the AE and control group, data from another group of subjects age-matched to the AE patients were analyzed. We found a very similar pattern in the comparison of functional connection networks in Figure S7. In Figure S8, we also showed that the differential connectivity of orbital superior frontal gyrus is not significantly correlated with age in this control group. [file 734893.f1.pdf]

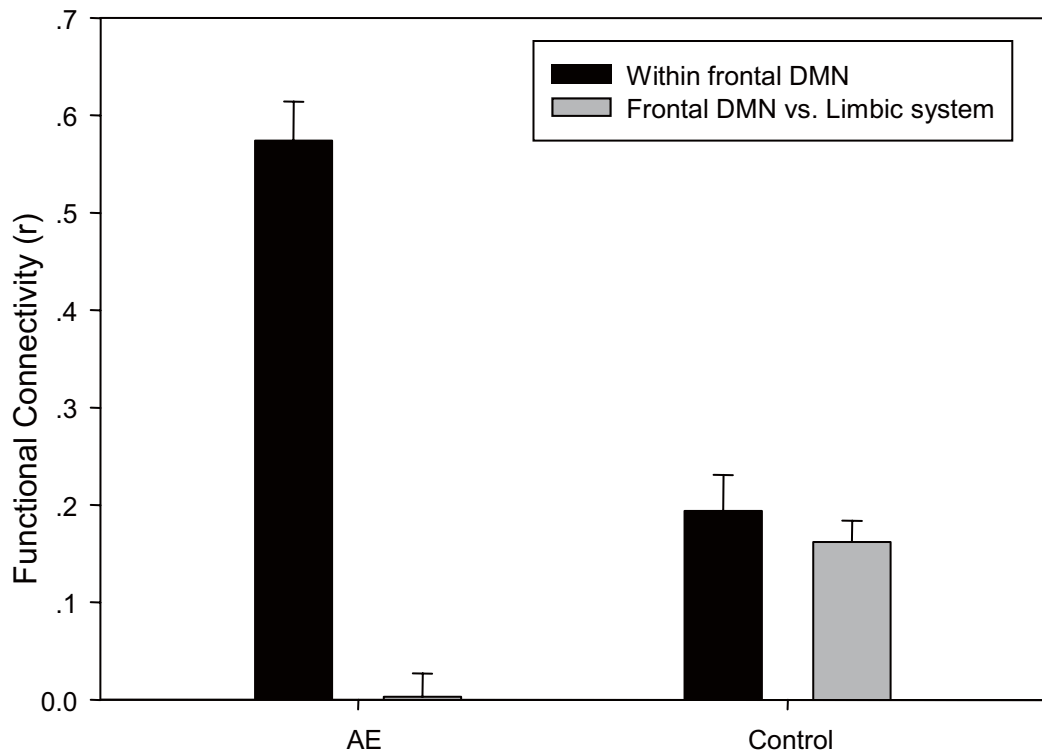

Supplement: Supplementary file 5 [file 734893.f5.pdf]

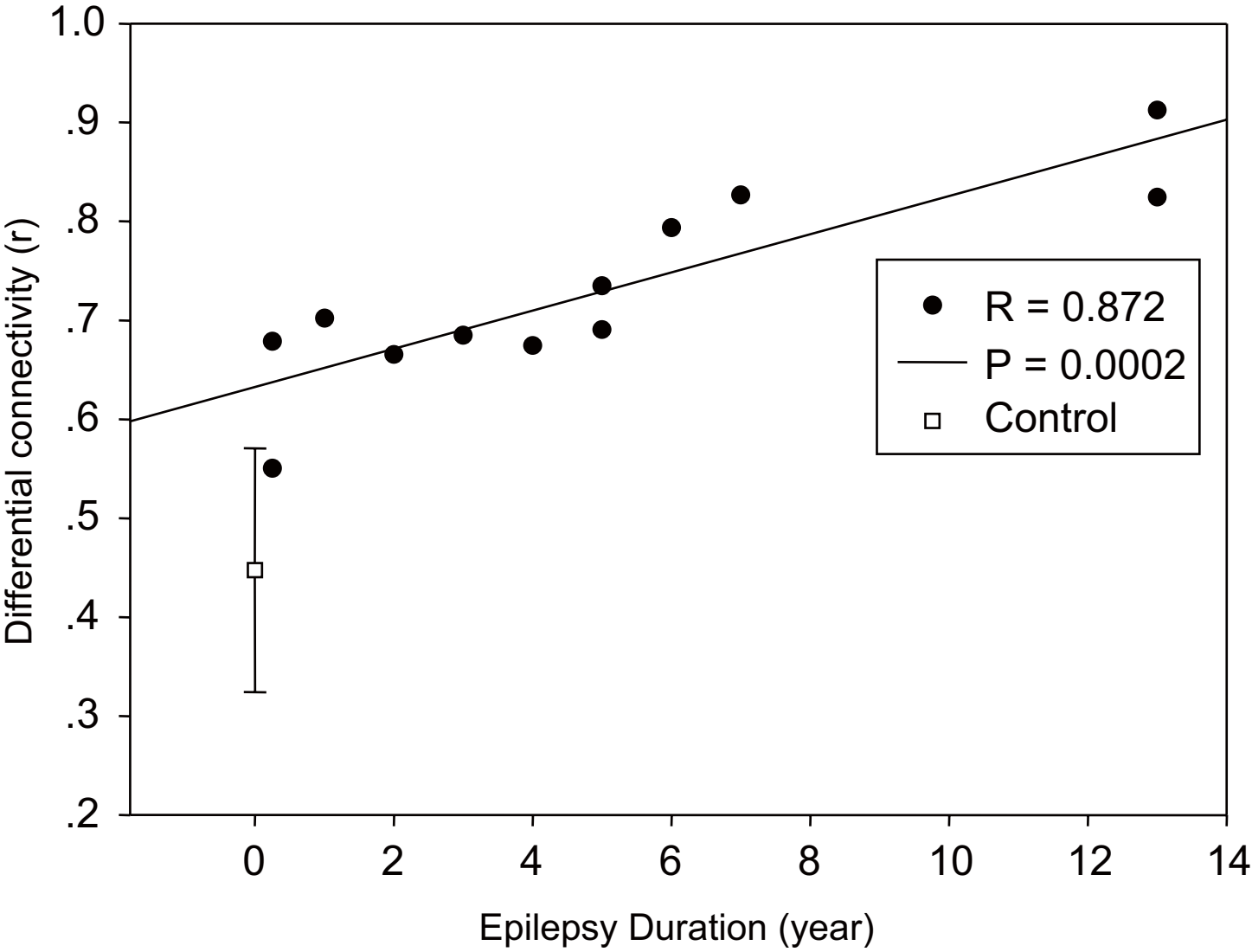

Supplement: Supplementary file 6 [file 734893.f6.pdf]

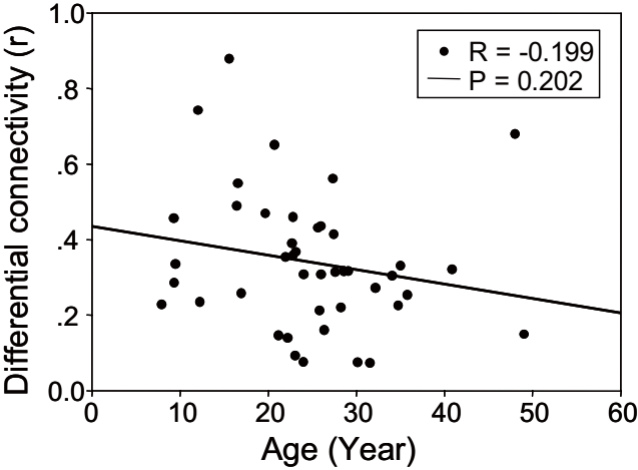

Supplement: Supplementary file 8 [file 734893.f8.pdf]
