# Supplementary material for: Altered Functional Connectivity within and between Brain Modules in Absence Epilepsy: A Resting-State Functional Magnetic Resonance Imaging Study
Source: Biomed Res Int. 2013 Sep 26;2013:734893. doi: 10.1155/2013/734893 (PMC3804038; doi:10.1155/2013/734893)
Supplement: Supplementary file 4 [file 734893.f4.pdf]

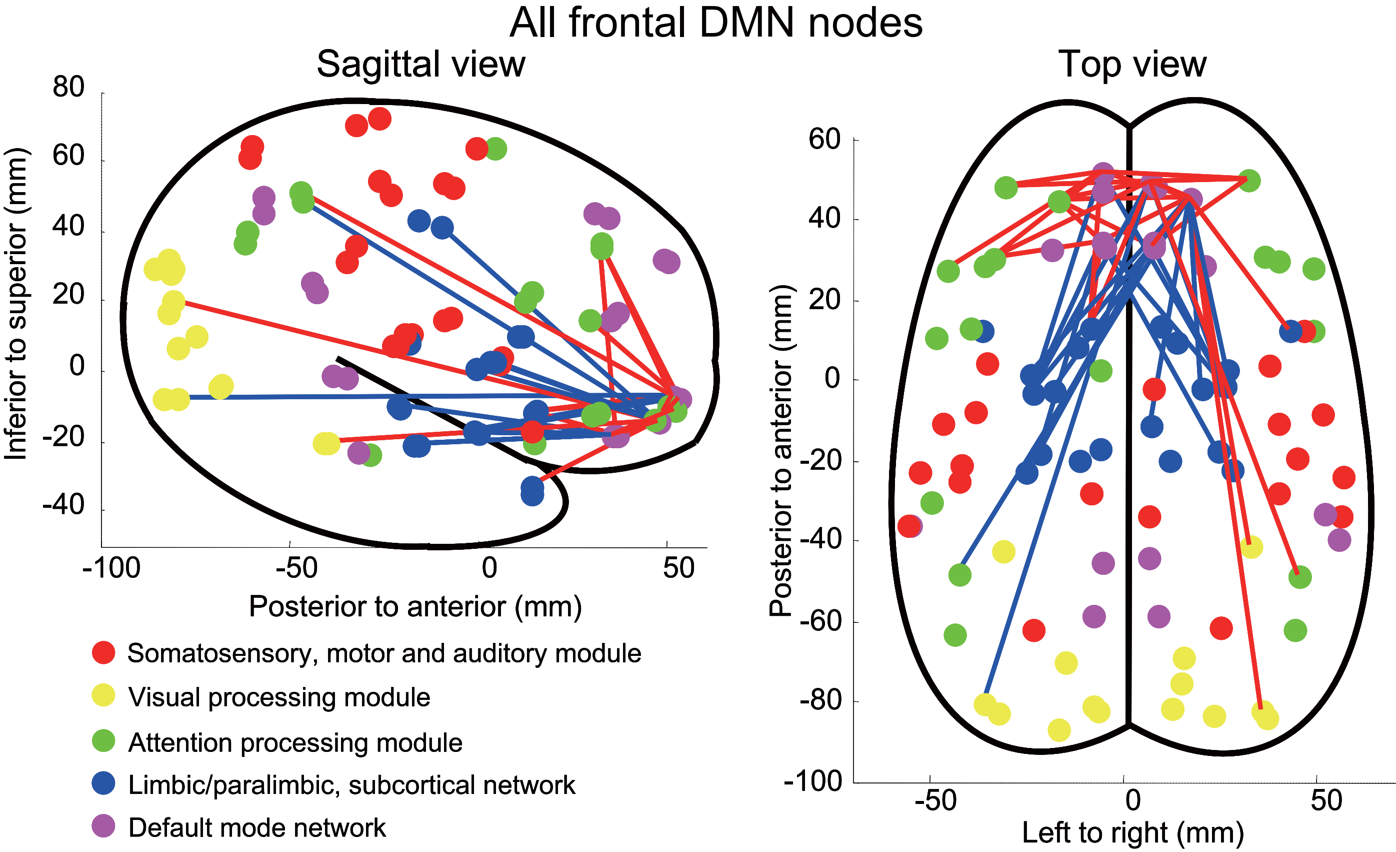
All frontal DMN nodes
Sagittal view
Top view
Inferior to superior (mm)
Posterior to anterior (mm)
Left to right (mm)
80
60
40
20
0
-20
-40
-100
-50
0
50
-60
-80
-100
Somatosensory, motor and auditory module
Visual processing module
Attention processing module
Limbic/paralimbic, subcortical network
Default mode network
