# Supplementary material for: Altered Functional Connectivity within and between Brain Modules in Absence Epilepsy: A Resting-State Functional Magnetic Resonance Imaging Study
Source: Biomed Res Int. 2013 Sep 26;2013:734893. doi: 10.1155/2013/734893 (PMC3804038; doi:10.1155/2013/734893)
Supplement: Supplementary file 7 [file 734893.f7.pdf]

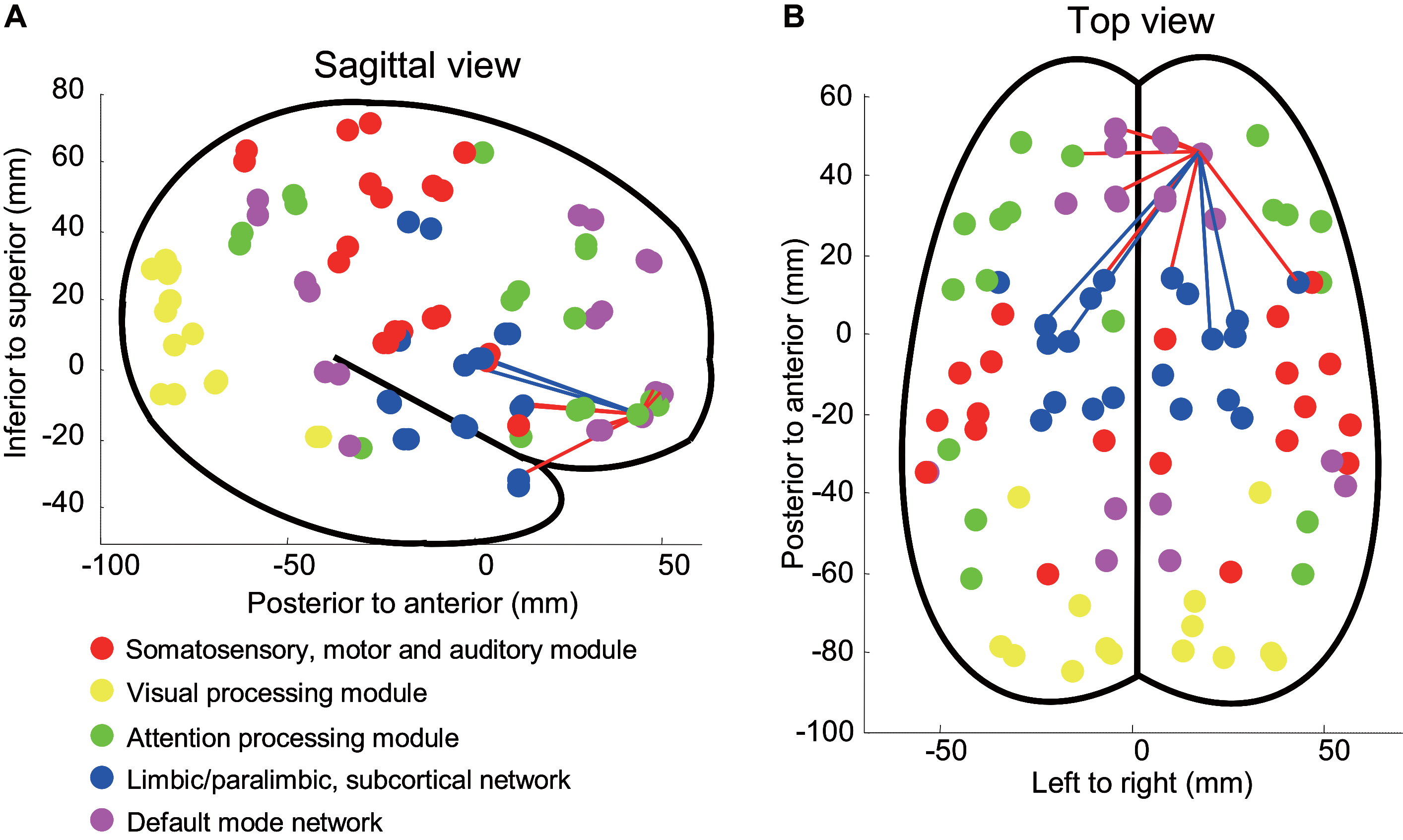
A
Sagittal view
Inferior to superior (mm)
80
60
40
20
0
-20
-40
-100
-50
0
50
Posterior to anterior (mm)
Somatosensory, motor and auditory module
Visual processing module
Attention processing module
Limbic/paralimbic, subcortical network
Default mode network
B
Top view
Posterior to anterior (mm)
60
40
20
0
-20
-40
-60
-80
-100
-50
0
50
Left to right (mm)
